# Effects of Postnatal Enriched Environment in a Model of Parkinson’s Disease in Adult Rats

**DOI:** 10.3390/ijms18020406

**Published:** 2017-02-14

**Authors:** Adel Jungling, Dora Reglodi, Zsofia Nozomi Karadi, Gabor Horvath, Jozsef Farkas, Balazs Gaszner, Andrea Tamas

**Affiliations:** Department of Anatomy, University of Pecs Medical School, Pecs 7624, Hungary; junglingadel@gmail.com (A.J.); dora.reglodi@aok.pte.hu (D.R.); zsofia.karadi@gmail.com (Z.N.K.); gabor.horvathmd@gmail.com (G.H.); jozsef.farkas@aok.pte.hu (J.F.); balazs.b.gaszner@aok.pte.hu (B.G.)

**Keywords:** postnatal, enriched environment, Parkinson’s disease, 6-OHDA, rat, substantia nigra, neuroprotection

## Abstract

Environmental enrichment is a widespread neuroprotective strategy during development and also in the mature nervous system. Several research groups have described that enriched environment in adult rats has an impact on the progression of Parkinson’s disease (PD). The aim of our present study was to examine the effects of early, postnatal environmental enrichment after 6-hydroxydopamine-induced (6-OHDA) lesion of the substantia nigra in adulthood. Newborn Wistar rats were divided into control and enriched groups according to their environmental conditions. For environmental enrichment, during the first five postnatal weeks animals were placed in larger cages and exposed to intensive complex stimuli. Dopaminergic cell loss, and hypokinetic and asymmetrical signs were evaluated after inducing PD with unilateral injections of 6-OHDA in three-month-old animals. Treatment with 6-OHDA led to a significant cell loss in the substantia nigra of control animals, however, postnatal enriched circumstances could rescue the dopaminergic cells. Although there was no significant difference in the percentage of surviving cells between 6-OHDA-treated control and enriched groups, the slightly less dopaminergic cell loss in the enriched group compared to control animals resulted in less severe hypokinesia. Our investigation is the first to provide evidence for the neuroprotective effect of postnatal enriched environment in PD later in life.

## 1. Introduction

Parkinson’s disease (PD) is a progressive degenerative disorder of the central nervous system. It is the second most common neurodegenerative disease and its prevalence increases with age. Symptoms of PD appear most often around the age of 60 [1,2]. Although the cause of the disease is still unknown, remarkable progress has been made to understand the underlying mechanisms [3]. Several genes, such as α-synuclein, parkin, PINK1 (phosphatase and tensin homologue-induced kinase 1), DJ1 (also known as Parkinson disease protein 7, PARK7 gene); and also environmental factors, like physical trauma, infections, and toxic effects have been identified to have a role in PD [4,5]. It is characterized by the death of the dopamin-producing (DAergic) neurons of the substantia nigra pars compacta [6] and the accumulation of Lewy bodies, the intracytoplasmic α-synuclein inclusions in the surviving neurons [7,8]. In consequence, the dopamine (DA) level of the nigrostriatal system decreases, which leads to disrupted connections to the thalamus and motor cortex [9]. Due to these changes of the nigrostriatal circuit, the most typical clinical signs of PD include motor symptoms, such as tremor, muscular rigidity, bradykinesia, and postural abnormalities. The aim of the currently available therapy is only to relieve symptoms by substituting the loss of DA, but all therapeutical options fail to avoid the progression of degeneration [10]. Several researchers focus on possible new neuroprotective agents that could prevent the DAergic cell loss [10,11,12,13,14,15]. In addition to pharmacological therapy, several studies have described that environmental factors can change the prevalence and outcome of PD [16]. In humans, physical exercise and sport in younger ages appear to be protective later [17,18,19].

Environmental enrichment has been shown to be neuroprotective in animal models of different brain pathologies. The beneficial effects of enriched circumstances were first described by Donald O. Hebb, when he observed that rats kept as pets performed better in problem solving, memory, and learning tasks [20]. Since these first findings, numerous studies have described the importance of environmental factors. Environmental enrichment is able to impact the development of the nervous system [21,22]. It is capable of increasing gliogenesis in cortical regions [23] and the number of oligodendrocytes [24]; it enhances synapse formation, and increases angiogenesis [25] and the thickness of cortex [26]. Environmental enrichment is a widespread neuroprotective strategy also in the mature nervous system. Our research group has shown that it can act as a protective factor against several types of harmful stimuli, like postnatal monosodium-glutamate toxicity [27], as well as neonatal and adult retina lesions [28,29]. In the last decade, impressive results have been obtained on the effects of enriched conditions in neurodegenerative diseases, such as Huntington’s disease, Alzheimer’s disease, amyotrophic lateral sclerosis, and Parkinson’s disease. The mechanisms underlying these protective effects include increasing the levels of several neurotrophic and neuroprotective factors, for example, brain-derived neurotrophic factor (BDNF), nerve growth factor (NGF), and glial cell-derived neurotrophic factor (GDNF) [30].

The most well-known rodent models of PD are created by 1-methyl-4-phenyl-1,2,3,6-tetrahydropyridine (MPTP) or the unilateral lesion of substantia nigra with 6-hydroxydopamine (6-OHDA) [31,32,33]. Studies on both MPTP [34,35] and 6-OHDA-induced [36,37,38] models have provided evidence for the protective effect of environmental enrichment in Parkinson’s disease. Although the application time of enriched conditions was different in each study, animals assigned to enriched environment showed increased resistance to MPTP insult, decreased loss of DAergic neurons, decreased dopamine transporter expression, and increased BDNF levels [30]. These results were found in adult, three to six-months-old animals, when they were exposed to enriched environment directly before, meanwhile, or shortly after inducing PD. It is well known that environmental factors in the early ages of life are very important in the development of the nervous system. Harmful stimuli, such as hypoxic and toxic lesions, but also positive effects can have consequences later in life [27,39,40,41], due to the extreme plasticity and regenerative capacity of the nervous system at this age. Based on the above, the aim of the present study was to investigate the effects of early, postnatal environmental enrichment on the functional and morphological changes in a model of Parkinson’s disease in adult rats.

## 2. Results

### 2.1. Evaluation of Behaviour

Behavioral parameters tested were focused on two groups of signs: hypokinetic and asymmetrical signs.

#### 2.1.1. Examination of Hypokinetic Signs

**Figure 1:**
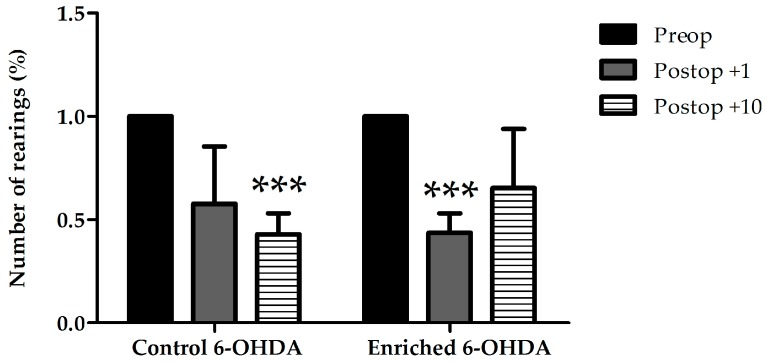
Rearing activity in 6-OHDA-treated control and enriched animal groups before (Preop), 1 day, and 10 days after the lesion (Postop +1, +10, respectively). In the graph the percentage of free rearings is shown compared to preoperative data of the same group. Values are given as mean ± S.E.M., where 100% = 1.0. *** *p* < 0.001 versus preoperative data of the same group.

Behavioral examinations showed hypokinesia due to the lesion of dopaminergic cells. We did not find any significant differences between the examined animal groups in case of resting time and the number of rearings against the wall with both limbs (data not shown). The decrease of the total number of free rearings indicates impaired motor function. In case of animals kept in standard environment (control) treated with 6-OHDA the total number of free rearings was significantly reduced on the 10th postoperative day when compared to preoperative data. A significant decrease could only be observed on the first postoperative day, and a recovery was visible after 10 days in enriched animals (Figure 1).

**Figure 2:**
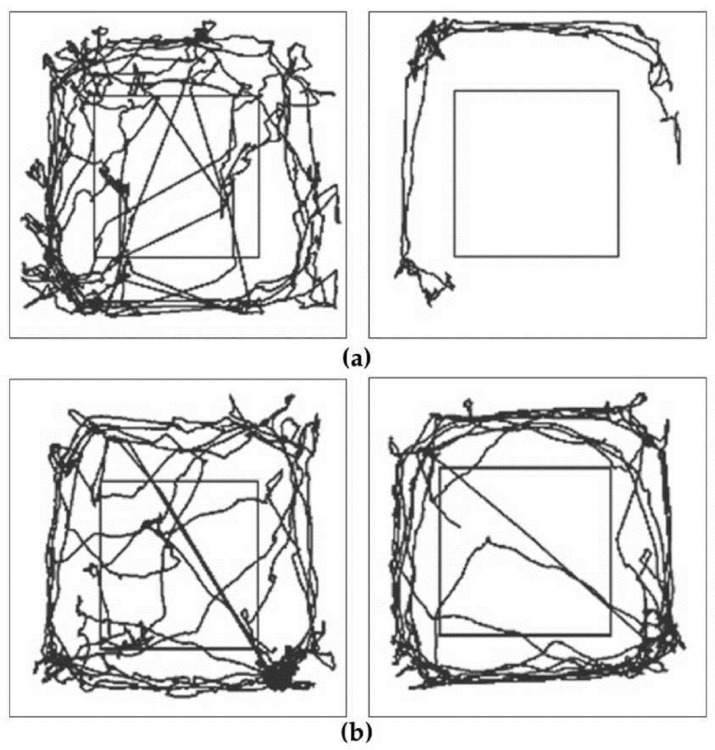
Distance covered in a representative 6-OHDA-treated control (**a**) and enriched animal (**b**); evaluated with Smart Junior program (Panlab, Harvard Apparatus, Barcelona, Spain). Movement of the animals was followed before (**left panels**) and 10 days after the operation (**right panels**).

**Figure 3:**
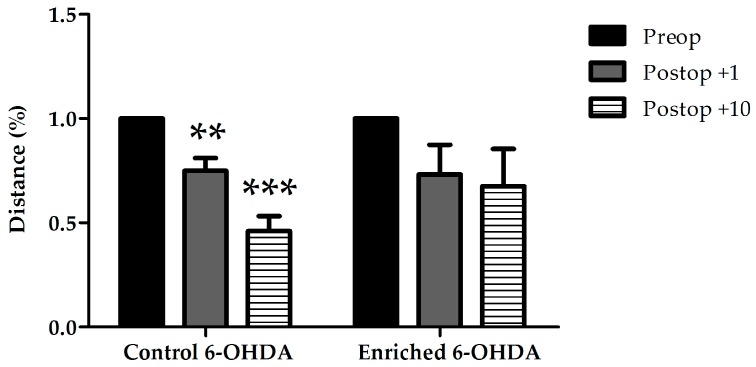
Distance covered in 6-OHDA-treated control and enriched animal groups before (Preop), 1 day, and 10 days after the lesion (Postop +1, +10, respectively). In the graph the percentage of distance covered is shown compared to preoperative data of the same group. Values are given as mean ± S.E.M., where 100% = 1.0. ** *p* < 0.01, *** *p* < 0.001 versus preoperative data of the same group.

Evaluation of resting time, time spent with slow movement, minimum and maximum velocity, measured by Smart Junior program, did not show significant alterations between control and enriched animals (data not shown). The distance covered by the animals was also measured (Figure 2 and Figure 3). Control 6-OHDA-treated animals moved significantly less on both examined days after the lesion. However, enriched animals showed a better performance: in this group the distance covered did not decrease significantly after 6-OHDA-induced lesion (Figure 2 and Figure 3).

**Figure 4:**
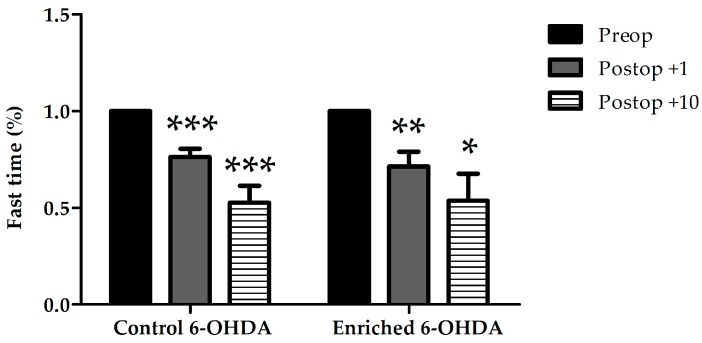
Fast time in 6-OHDA-treated control and enriched animal groups before (Preop), 1 day, and 10 days after the lesion (Postop +1, +10, respectively). In the graph the percentage of fast time is shown compared to preoperative data of the same group. Values are given as mean ± S.E.M., where 100% = 1.0. * *p* < 0.05, ** *p* < 0.01, *** *p* < 0.001 versus preoperative data of the same group.

Time spent with fast movement (fast time) was also evaluated (Figure 4). We could observe a significant decrease of movements in both control and enriched animal groups after the operation at both examined postoperative days. However, we could not find statistical difference between the control and enriched groups.

#### 2.1.2. Examination of Asymmetrical Signs

**Figure 5:**
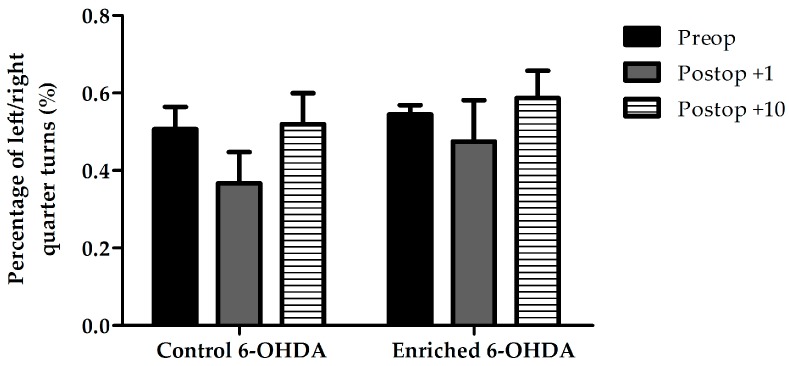
Percentage of left/right quarter turns in 6-OHDA-treated control and enriched animal groups before (Preop), 1 day, and 10 days after the lesion (Postop +1, +10, respectively). Values are given as mean ± S.E.M., where 100% = 1.0.

No significant differences were found between the examined groups regarding the percentage of left/right quarter turns (Figure 5) and the number of rearings against the wall with left or right limb, indicating the lack of asymmetrical behavioral signs.

### 2.2. Tyrosine-Hydroxylase (TH)-Immunohistochemistry

**Figure 6:**
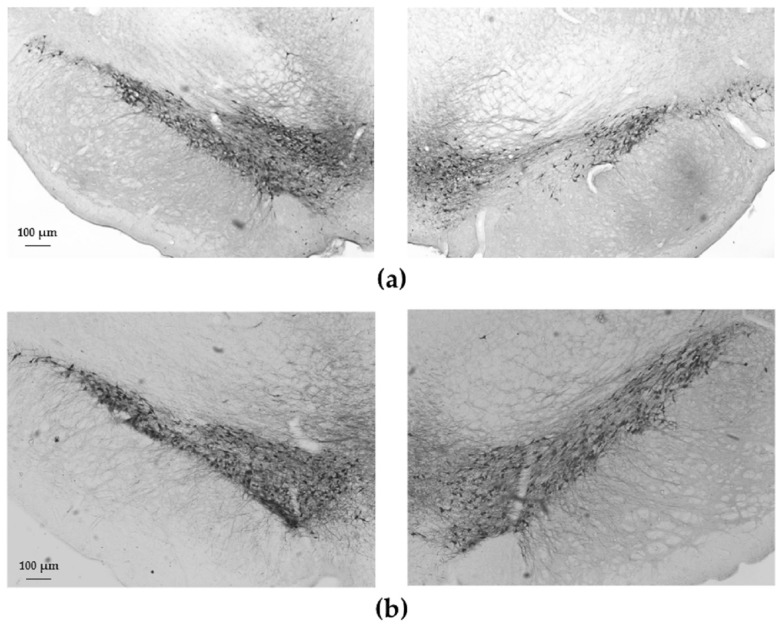
Tyrosine-hydroxylase (TH)-immunoreactivity in a representative 6-OHDA-treated control (**a**); and enriched animal (**b**). Sections were photographed from the substantia nigra (**a**,**b**) from both the lesioned (**right panels**) and the contralateral sides (**left panels**).

**Figure 7:**
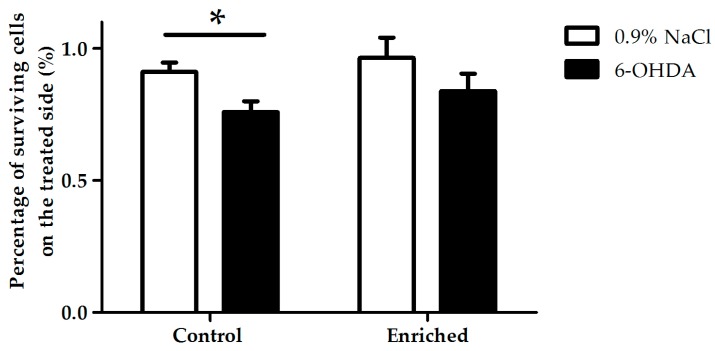
Percentage of TH-immunoreactive surviving cells on the treated side, compared to control side in saline (0.9% NaCl) and 6-OHDA-treated animals of control and enriched groups. Data are given as mean ± S.E.M., where 100% = 1.0. * *p* < 0.05 versus control 0.9% NaCl-treated group.

Our morphometric studies revealed a significant cell loss in the substantia nigra pars compacta in 6-OHDA-treated animals of the control group compared to saline-treated (0.9% NaCl) animals of the same group (Figure 6). In contrast, in the case of enriched animals, 6-OHDA did not cause significant dopaminergic cell loss compared to the saline-treated enriched group. The percentage of surviving cells of the lesioned side was 76% ± 4% of the intact side in the control group, while it was 84% ± 6% in the enriched group. There was no significant difference between control and enriched groups (Figure 7).

## 3. Discussion

In the present study, we provided evidence for the protective effect of early, postnatal environmental enrichment in Parkinson’s disease in adult rats. As early life events have critical importance in the development of the nervous system, numerous research studies have investigated this crucial period. It has been shown that effects in postnatal life can have long-term consequences. Negative effects, injuries, and environmental challenges occurring in this period can be considered as etiological factors in neuropsychiatric and neurodegenerative diseases [42,43]. Perinatal asphyxia and hypoxia can lead to permanent brain injuries causing seizures, cerebral palsy, and learning and behavioral deficits [44,45]. Cerebellar histomorphology of rats also change after insufficient oxygenization [46]. Exposure to different types of toxic materials, such as excitotoxic agents, pesticides, and ethanol can also induce neural disorders [47,48,49,50]. Although many factors contribute to increased vulnerability to neuropsychiatric disorders, numerous studies have shown that postnatal stress is a major factor both in rodents [51,52,53] and humans [54,55,56].

Positive environmental factors are proven to counteract effects of neuronal injuries. One of these well-studied factors is the enriched environment. Under experimental conditions environmental enrichment refers to housing conditions when complex motor, sensory, and cognitive stimulation is provided compared to a standard environment [30]. Numerous investigations provide evidence that enriched circumstances have protective effects against neural lesions caused by ischemic [57,58,59], toxic [27,60,61], and traumatic injuries [62,63,64]. Several research groups have described that enriched environment has an impact on the severity of symptoms and the progression of Parkinson’s disease. Animals exposed to enriched circumstances are shown to be less vulnerable to MPTP insult. A higher number of surviving DAergic neurons, increased expression of GDNF and BDNF in the striatum, and decreased levels of dopamine transporter (DAT) were found in enriched animals. DAT plays a role in mediating the deleterious effects of MPTP, thus its decrease contributes to the resistance against MPTP in enriched animals [34,35]. Furthermore, it has been described that rats housed in enriched conditions showed significantly improved motor performance associated with a decreased loss of DAergic neurons, dopamine and its metabolites in the striatum in 6-OHDA-induced rat models of PD. An increased number of GFAP-positive (glial fibrillary acidic protein) cells was also found in enriched animals [36,37,38]. These changes can contribute to the neuroprotective effect of environmental enrichment.

In the case of these previous experimental setups, environmental enrichment was applied directly before and/or shortly after the induction of Parkinson’s disease, in three to six-month-old animals, for different time-periods (four, six, or eight weeks prior to lesion, three weeks before and three weeks after the lesion). However, there is no data about the long-term effects of postnatal environmental enrichment so far. In our present study, we aimed to assess the long-term effects of an early enrichment. After birth, during the first five postnatal weeks pups were placed in larger cages, supplemented with different toys and subjects which provided complex stimuli, and their daily change offered continuous novelty for cognitive stimuli. In three-month-old animals after inducing PD we evaluated the dopaminergic cell loss and motor signs. Treatment with 6-OHDA led to a significant cell loss in the substantia nigra in control animals, however, postnatal enriched circumstances could rescue dopaminergic cells. Although there was no significant difference in the percentage of surviving cells between 6-OHDA-treated control and enriched groups, the slightly less dopaminergic cell loss in the enriched group compared to control animals resulted in less severe motor signs. This phenomenon showing that slight difference in dopaminergic cell number can lead to ameliorated behavioral recovery, was previously observed by our research group with another neuroprotective treatment strategy [16]. During evaluation of the number of free rearings we found reduced rearing activity after 6-OHDA injections in both groups. However, enriched animals showed a recovery on the tenth day after the acute decrease on the first postoperative day, suggesting a better ability for compensation in enriched animals. Regarding the distance travelled, enriched animals performed clearly better, as there was no significant impairment in their movement. The time spent with fast movement dropped significantly both in control and enriched groups. We suggest that the lack of asymmetrical behavioral signs is due to the low extent of the injury. It is well known that only more that 50% loss of the DAergic neurons leads to marked asymmetrical symptoms in this model, and the maximal lesion in our present study was only 24% [15,16].

Although we did not find dramatic protective effects of the postnatal enriched environment, our results complete previous findings and provide further evidence for its neuroprotective effect. The extent of protection in the case of early environmental factors are never as strong as with therapeutic agents, but we have to take environmental factors into consideration and encourage the discovery of possible ways of prevention. Human studies also revealed that regular physical activity and exercise result in lower risk of PD and alleviates motor symptoms of the disease [17]. Correspondingly aerobic exercise causes immediate improvement of gait and balance in PD patients [18].

Our findings draw further attention to the importance of postnatal environmental factors and their capacity to prevent and modify symptoms of neurodegenerative diseases in adulthood.

## 4. Materials and Methods

Wistar rats from a local colony were used for our experiments (*n* = 29). Animal housing, care, and application of experimental procedures were in accordance with institutional guidelines under approved protocols (No: BA02/2000-15024/2011, University of Pecs following the European Community Council directive). We paid special attention to conduct these experiments on animals that were born exactly at the same time, to avoid any environmental effects other than our enriched/non-enriched environment. Food and water were available ad libitum and rats were kept under a 12-h light-dark cycle.

Similarly to our earlier studies [27] animals were divided into control (*n* = 16) and enriched (*n* = 13) groups according to their environmental conditions. Animals of the control group were kept under regular conditions (cages with 43 cm × 30 cm × 20 cm). For environmental enrichment, during the first five postnatal weeks we placed pups in larger cages (88 cm × 50 cm × 44 cm) supplemented with toys, objects, running tunnels, and rotating rods of different shapes, sizes, and materials. Half of the toys were changed daily. This way the rats of the enriched group were continuously exposed to intensive complex sensory, motor, and also cognitive stimuli. After this five-week-long period these animals were also kept under regular circumstances until adulthood [41].

At the age of three months Parkinson’s disease was induced similarly to our previous studies [16]. One group of the animals was treated with unilateral injections of 2 µL 6-OHDA (5 µg/1 µL, Sigma, Budapest, Hungary) into the left substantia nigra (from bregma point: 5.5 mm posteriorly, 2.5 mm left, and 8 mm ventrally). The other group of the animals received the same volume of physiological saline (0.9% NaCl) to the same location. The injections were given with a Hamilton needle, each time over a 2-min period, and the needle was left in place for another minute. The right side of the animals was always left untreated, serving as control side.

Behavioral experiments were done before the injury, and to assess acute behavioral deficits and the degree of recovery we repeated our measurements 1 and 10 days after the operation. For testing, rats were placed into a 42.5 cm × 42.5 cm box, with 50 cm high walls around. Each rat was video-recorded for 5 min. We observed two groups of signs: hypokinetic (resting time, number of free rearings without reaching the wall, as well as number of rearings against the wall with both limbs) and asymmetrical signs (such as the number of left and right quarter turns and rearing against the wall using only one limb). The environment and the treatment of the animals were not given to the investigator who evaluated these signs.

Open field application of the Smart Junior v1.0.07 video tracking program (Panlab, Harvard Apparatus, Barcelona, Spain) was also used to further analyze the movements of the animals. The following parameters were measured by this program: total distance covered (cm), resting time (s), time spent with slow (when velocity was lower than 10 cm/s) and fast movement (when velocity was higher than 100 cm/s), and minimum and maximum velocity. Data in the behavioral tests are expressed as percentage of each sign ± S.E.M. compared to preoperative data of the same group. Results were compared using two sample t-tests, differences were considered significant at *p* < 0.05. In the case of significant differences, we also calculated the effect size, as the difference between the means of two groups (Cohen’s d). For all found differences the effect sizes were large (Cohen’s d values: range between 1.23 and 2.8).

Rats were intracardially perfused with 4% paraformaldehyde after the behavioral tests. Fifty-micrometer-thick vibrotome sections from the mesencephalon were made in the frontal plane (between 4.8 and 6.3 mm posterior from bregma point), and stained with TH mouse monoclonal antibody (1:1000, Sigma). This is the most commonly used marker enzyme for dopaminergic neurons. Sections were incubated with the antiserum for 48 h at 4 °C, followed by incubation in the secondary biotinylated antibody (Jackson ImmunoResearch Laboratories Inc., West Groove, PA, USA) for 1 h and in an avidin-biotinylated-peroxidase complex, according to the instructions of the ABC kit (Vector Laboratories Inc., Burlingame, CA, USA). Digital photomicrographs were taken with a Nikon FXA photomicroscope (Nikon Corp., Tokyo, Japan) under 40× magnification attached to a digital camera (Spot RT Color camera) [16].

In each 50-µm-section on both sides of the substantia nigra pars compacta TH-positive cells with a visible nucleus were counted using ImageJ software (version 1.50i, National Institutes of Health, Bethesda, MD, USA). Cell count was always done by an investigator unaware of the group of the animals. The percentage of TH-positive cells on the lesioned (left) side was compared to the control, undamaged side in each section. Data are expressed as mean percentage of surviving cells on the lesioned side ± S.E.M. Results were compared using two-way ANOVA test followed by Fischer‘s post hoc analysis; differences were considered significant at *p* < 0.05.

## 5. Conclusions

In summary our present results are the first to provide evidence for the neuroprotective effect of postnatal enriched environment in Parkinson’s disease in adult rats. We showed that enriched housing conditions in early life can rescue the dopaminergic cells after 6-OHDA treatment in adulthood. In addition, less nigral cell loss in enriched animals led to less severe hypokinetic symptoms.
